# Developing a survey to measure nursing students’ knowledge, attitudes and beliefs, influences, and willingness to be involved in Medical Assistance in Dying (MAiD): a mixed method modified e-Delphi study

**DOI:** 10.1186/s12912-024-01984-z

**Published:** 2024-05-14

**Authors:** Jocelyn Schroeder, Barbara Pesut, Lise Olsen, Nelly D. Oelke, Helen Sharp

**Affiliations:** 1 School of Health and Human Services, Selkirk College, Castlegar, BC Canada; 2 School of Nursing, University of British Columbia Okanagan, Kelowna, BC Canada

**Keywords:** Medical assistance in dying (MAiD), Euthanasia, Canada, End of life care, Student nurses, Survey, Nursing education

## Abstract

**Background:**

Medical Assistance in Dying (MAiD) was legalized in Canada in 2016. Canada’s legislation is the first to permit Nurse Practitioners (NP) to serve as independent MAiD assessors and providers. Registered Nurses’ (RN) also have important roles in MAiD that include MAiD care coordination; client and family teaching and support, MAiD procedural quality; healthcare provider and public education; and bereavement care for family. Nurses have a right under the law to conscientious objection to participating in MAiD. Therefore, it is essential to prepare nurses in their entry-level education for the practice implications and moral complexities inherent in this practice. Knowing what nursing students think about MAiD is a critical first step. Therefore, the purpose of this study was to develop a survey to measure nursing students’ knowledge, attitudes and beliefs, influences, and willingness to be involved in MAiD in the Canadian context.

**Methods:**

The design was a mixed-method, modified e-Delphi method that entailed item generation from the literature, item refinement through a 2 round survey of an expert faculty panel, and item validation through a cognitive focus group interview with nursing students. The settings were a University located in an urban area and a College located in a rural area in Western Canada.

**Results:**

During phase 1, a 56-item survey was developed from existing literature that included demographic items and items designed to measure experience with death and dying (including MAiD), education and preparation, attitudes and beliefs, influences on those beliefs, and anticipated future involvement. During phase 2, an expert faculty panel reviewed, modified, and prioritized the items yielding 51 items. During phase 3, a sample of nursing students further evaluated and modified the language in the survey to aid readability and comprehension. The final survey consists of 45 items including 4 case studies.

**Discussion:**

Systematic evaluation of knowledge-to-date coupled with stakeholder perspectives supports robust survey design. This study yielded a survey to assess nursing students’ attitudes toward MAiD in a Canadian context.

**Conclusion:**

The survey is appropriate for use in education and research to measure knowledge and attitudes about MAiD among nurse trainees and can be a helpful step in preparing nursing students for entry-level practice.

**Supplementary Information:**

The online version contains supplementary material available at 10.1186/s12912-024-01984-z.

## Background

Medical Assistance in Dying (MAiD) is permitted under an amendment to Canada’s Criminal Code which was passed in 2016 [1]. MAiD is defined in the legislation as both self-administered and clinician-administered medication for the purpose of causing death. In the 2016 Bill C-14 legislation one of the eligibility criteria was that an applicant for MAiD must have a reasonably foreseeable natural death although this term was not defined. It was left to the clinical judgement of MAiD assessors and providers to determine the time frame that constitutes reasonably foreseeable [2]. However, in 2021 under Bill C-7, the eligibility criteria for MAiD were changed to allow individuals with irreversible medical conditions, declining health, and suffering, but whose natural death was not reasonably foreseeable, to receive MAiD [3]. This population of MAiD applicants are referred to as Track 2 MAiD (those whose natural death is foreseeable are referred to as Track 1). Track 2 applicants are subject to additional safeguards under the 2021 C-7 legislation.

Three additional proposed changes to the legislation have been extensively studied by Canadian Expert Panels (Council of Canadian Academics [CCA]) [4–6] First, under the legislation that defines Track 2, individuals with mental disease as their sole underlying medical condition may apply for MAiD, but implementation of this practice is embargoed until March 2027 [4]. Second, there is consideration of allowing MAiD to be implemented through advanced consent. This would make it possible for persons living with dementia to receive MAID after they have lost the capacity to consent to the procedure [5]. Third, there is consideration of extending MAiD to mature minors. A mature minor is defined as “a person under the age of majority…and who has the capacity to understand and appreciate the nature and consequences of a decision” ([6] p. 5). In summary, since the legalization of MAiD in 2016 the eligibility criteria and safeguards have evolved significantly with consequent implications for nurses and nursing care. Further, the number of Canadians who access MAiD shows steady increases since 2016 [7] and it is expected that these increases will continue in the foreseeable future.

Nurses have been integral to MAiD care in the Canadian context. While other countries such as Belgium and the Netherlands also permit euthanasia, Canada is the first country to allow Nurse Practitioners (Registered Nurses with additional preparation typically achieved at the graduate level) to act independently as assessors and providers of MAiD [1]. Although the role of Registered Nurses (RNs) in MAiD is not defined in federal legislation, it has been addressed at the provincial/territorial-level with variability in scope of practice by region [8, 9]. For example, there are differences with respect to the obligation of the nurse to provide information to patients about MAiD, and to the degree that nurses are expected to ensure that patient eligibility criteria and safeguards are met prior to their participation [10]. Studies conducted in the Canadian context indicate that RNs perform essential roles in MAiD care coordination; client and family teaching and support; MAiD procedural quality; healthcare provider and public education; and bereavement care for family [9, 11]. Nurse practitioners and RNs are integral to a robust MAiD care system in Canada and hence need to be well-prepared for their role [12].

Previous studies have found that end of life care, and MAiD specifically, raise complex moral and ethical issues for nurses [13–16]. The knowledge, attitudes, and beliefs of nurses are important across practice settings because nurses have consistent, ongoing, and direct contact with patients who experience chronic or life-limiting health conditions. Canadian studies exploring nurses’ moral and ethical decision-making in relation to MAiD reveal that although some nurses are clear in their support for, or opposition to, MAiD, others are unclear on what they believe to be good and right [14]. Empirical findings suggest that nurses go through a period of moral sense-making that is often informed by their family, peers, and initial experiences with MAID [17, 18]. Canadian legislation and policy specifies that nurses are not required to participate in MAiD and may recuse themselves as conscientious objectors with appropriate steps to ensure ongoing and safe care of patients [1, 19]. However, with so many nurses having to reflect on and make sense of their moral position, it is essential that they are given adequate time and preparation to make an informed and thoughtful decision before they participate in a MAID death [20, 21].

It is well established that nursing students receive inconsistent exposure to end of life care issues [22] and little or no training related to MAiD [23]. Without such education and reflection time in pre-entry nursing preparation, nurses are at significant risk for moral harm. An important first step in providing this preparation is to be able to assess the knowledge, values, and beliefs of nursing students regarding MAID and end of life care. As demand for MAiD increases along with the complexities of MAiD, it is critical to understand the knowledge, attitudes, and likelihood of engagement with MAiD among nursing students as a baseline upon which to build curriculum and as a means to track these variables over time.

## Methods

### Aim, design, and setting

The aim of this study was to develop a survey to measure nursing students’ knowledge, attitudes and beliefs, influences, and willingness to be involved in MAiD in the Canadian context. We sought to explore both their willingness to be involved in the registered nursing role and in the nurse practitioner role should they chose to prepare themselves to that level of education. The design was a mixed-method, modified e-Delphi method that entailed item generation, item refinement through an expert faculty panel [24–26], and initial item validation through a cognitive focus group interview with nursing students [27]. The settings were a University located in an urban area and a College located in a rural area in Western Canada.

### Participants

A panel of 10 faculty from the two nursing education programs were recruited for Phase 2 of the e-Delphi. To be included, faculty were required to have a minimum of three years of experience in nurse education, be employed as nursing faculty, and self-identify as having experience with MAiD. A convenience sample of 5 fourth-year nursing students were recruited to participate in Phase 3. Students had to be in good standing in the nursing program and be willing to share their experiences of the survey in an online group interview format.

### Procedures

The modified e-Delphi was conducted in 3 phases: Phase 1 entailed item generation through literature and existing survey review. Phase 2 entailed item refinement through a faculty expert panel review with focus on content validity, prioritization, and revision of item wording [25]. Phase 3 entailed an assessment of face validity through focus group-based cognitive interview with nursing students.

### Phase I. Item generation through literature review

The goal of phase 1 was to develop a bank of survey items that would represent the variables of interest and which could be provided to expert faculty in Phase 2. Initial survey items were generated through a literature review of similar surveys designed to assess knowledge and attitudes toward MAiD/euthanasia in healthcare providers; Canadian empirical studies on nurses’ roles and/or experiences with MAiD; and legislative and expert panel documents that outlined proposed changes to the legislative eligibility criteria and safeguards. The literature review was conducted in three online databases: CINAHL, PsycINFO, and Medline. Key words for the search included *nurses*, *nursing students*, *medical students*, *NPs, MAiD*, *euthanasia*, *assisted death*, and *end-of-life care*. Only articles written in English were reviewed. The legalization and legislation of MAiD is new in many countries; therefore, studies that were greater than twenty years old were excluded, no further exclusion criteria set for country.

Items from surveys designed to measure similar variables in other health care providers and geographic contexts were placed in a table and similar items were collated and revised into a single item. Then key variables were identified from the empirical literature on nurses and MAiD in Canada and checked against the items derived from the surveys to ensure that each of the key variables were represented. For example, conscientious objection has figured prominently in the Canadian literature, but there were few items that assessed knowledge of conscientious objection in other surveys and so items were added [15, 21, 28, 29]. Finally, four case studies were added to the survey to address the anticipated changes to the Canadian legislation. The case studies were based upon the inclusion of mature minors, advanced consent, and mental disorder as the sole underlying medical condition. The intention was to assess nurses’ beliefs and comfort with these potential legislative changes.

### Phase 2. Item refinement through expert panel review

The goal of phase 2 was to refine and prioritize the proposed survey items identified in phase 1 using a modified e-Delphi approach to achieve consensus among an expert panel [26]. Items from phase 1 were presented to an expert faculty panel using a Qualtrics (Provo, UT) online survey. Panel members were asked to review each item to determine if it should be: included, excluded or adapted for the survey. When adapted was selected faculty experts were asked to provide rationale and suggestions for adaptation through the use of an open text box. Items that reached a level of 75% consensus for either inclusion or adaptation were retained [25, 26]. New items were categorized and added, and a revised survey was presented to the panel of experts in round 2. Panel members were again asked to review items, including new items, to determine if it should be: included, excluded, or adapted for the survey. Round 2 of the modified e-Delphi approach also included an item prioritization activity, where participants were then asked to rate the importance of each item, based on a 5-point Likert scale (low to high importance), which De Vaus [30] states is helpful for increasing the reliability of responses. Items that reached a 75% consensus on inclusion were then considered in relation to the importance it was given by the expert panel. Quantitative data were managed using SPSS (IBM Corp).

### Phase 3. Face validity through cognitive interviews with nursing students

The goal of phase 3 was to obtain initial face validity of the proposed survey using a sample of nursing student informants. More specifically, student participants were asked to discuss how items were interpreted, to identify confusing wording or other problematic construction of items, and to provide feedback about the survey as a whole including readability and organization [31–33]. The focus group was held online and audio recorded. A semi-structured interview guide was developed for this study that focused on clarity, meaning, order and wording of questions; emotions evoked by the questions; and overall survey cohesion and length was used to obtain data (see Supplementary Material 2 for the interview guide). A prompt to “think aloud” was used to limit interviewer-imposed bias and encourage participants to describe their thoughts and response to a given item as they reviewed survey items [27]. Where needed, verbal probes such as “could you expand on that” were used to encourage participants to expand on their responses [27]. Student participants’ feedback was collated verbatim and presented to the research team where potential survey modifications were negotiated and finalized among team members. Conventional content analysis [34] of focus group data was conducted to identify key themes that emerged through discussion with students. Themes were derived from the data by grouping common responses and then using those common responses to modify survey items.

## Results

Ten nursing faculty participated in the expert panel. Eight of the 10 faculty self-identified as female. No faculty panel members reported conscientious objector status and ninety percent reported general agreement with MAiD with one respondent who indicated their view as “unsure.” Six of the 10 faculty experts had 16 years of experience or more working as a nurse educator.

Five nursing students participated in the cognitive interview focus group. The duration of the focus group was 2.5 h. All participants identified that they were born in Canada, self-identified as female (one preferred not to say) and reported having received some instruction about MAiD as part of their nursing curriculum. See Tables 1 and 2 for the demographic descriptors of the study sample. Study results will be reported in accordance with the study phases. See Fig. 1 for an overview of the results from each phase.

**Table 1:** Demographics of study sample. Characteristics of faculty participants

Sample Characteristics	*n*	*%*	*M*	*SD*
Age			50.11	6.99
Gender identity:				
Male	2	20		
Female	8	80		
Other	0	0		
Prefer not to say	0	0		
Sex assigned at birth:				
Male	2	20		
Female	8	80		
Other	0	0		
Prefer not to say	0	0		
Number of years worked as an educator			15.7	7.96
Level of agreement with MAiD:				
Agree	9	90		
Disagree	0	0		
Unsure	1	10		
Prefer not to say	0	0		
Identify as contentious objector to MAiD:				
Yes	0	0		
No	9	90		
Unsure	1	10		
Prefer not to say	0	0

**Table 2:** Demographics of study sample. Characteristics of student participants

Sample Characteristics	*n*	*%*
Age:		
Less than 20	0	0
20-29	2	40
30-39	1	20
40-49	2	40
Gender identity:		
Female	4	80
Other	0	0
Prefer not to say	1	20
Sex assigned at birth:		
Female	4	80
Other	0	0
Prefer not to say	1	20
Country primarily raised in:		
Canada	5	100
Religious and/or spiritual:		
Religious but not spiritual	0	0
Spiritual but not religious	3	60
Religious and spiritual	1	20
Neither religious nor spiritual	1	20
Prefer not to say	0	0
Importance of religion and/or spirituality:*		
Very unimportant	0	0
Unimportant	0	0
Neutral	1	25
Important	3	75
Very important	0	
Prefer not to say *did not record for 5^th^ participant	0	
Cared for a patient who has died during the period of time caring for them		
Yes	4	80
No	1	20
Discussed or been involved with discussions with a patient regarding end-of-life issues		
Yes	4	80
No	1	20
Cared for a patient who has requested MAiD		
Yes	4	80
No	1	20
Cared for a patient who later received MAiD		
Yes	3	60
No	1	20
Prefer not to say	1	20
Been in the room when a patient received MAiD		
Yes	4	80
No	1	20

^*^Missing data from one participant

**Figure:**
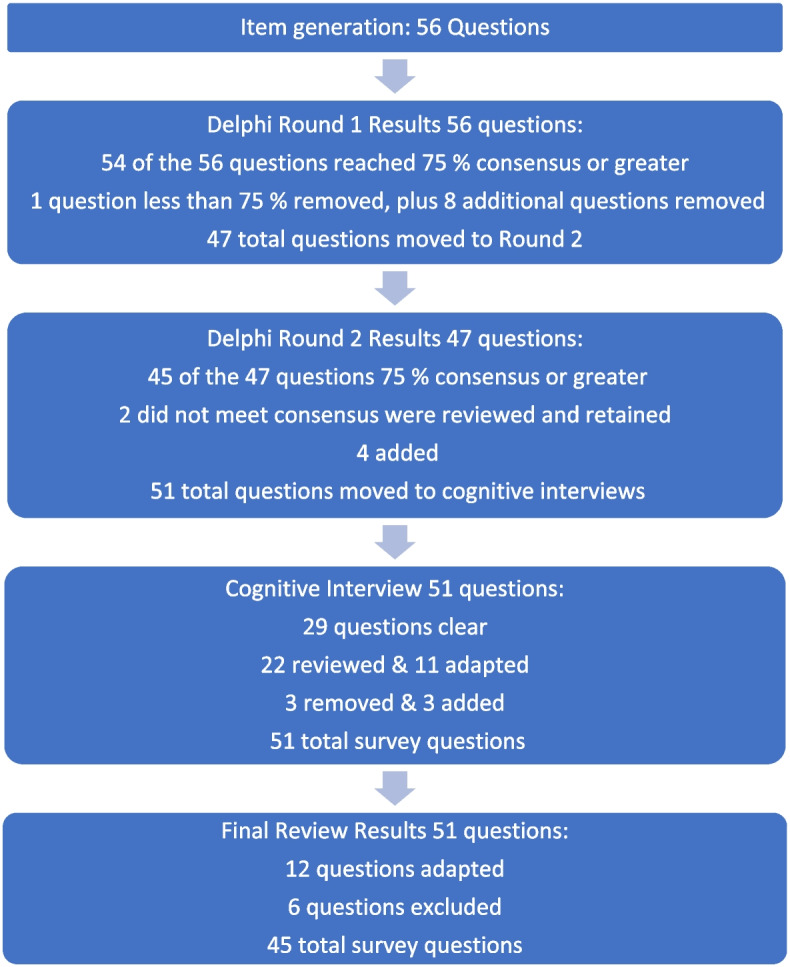
**Fig. 1** Overview of survey development findings

### Phase 1: survey item generation

Review of the literature identified that no existing survey was available for use with nursing students in the Canadian context. However, an analysis of themes across qualitative and quantitative studies of physicians, medical students, nurses, and nursing students provided sufficient data to develop a preliminary set of items suitable for adaptation to a population of nursing students.

Four major themes and factors that influence knowledge, attitudes, and beliefs about MAiD were evident from the literature: (i) endogenous or individual factors such as age, gender, personally held values, religion, religiosity, and/or spirituality [35–42], (ii) experience with death and dying in personal and/or professional life [35, 40, 41, 43–45], (iii) training including curricular instruction about clinical role, scope of practice, or the law [23, 36, 39], and (iv) exogenous or social factors such as the influence of key leaders, colleagues, friends and/or family, professional and licensure organizations, support within professional settings, and/or engagement in MAiD in an interdisciplinary team context [9, 35, 46].

Studies of nursing students also suggest overlap across these categories. For example, value for patient autonomy [23] and the moral complexity of decision-making [37] are important factors that contribute to attitudes about MAiD and may stem from a blend of personally held values coupled with curricular content, professional training and norms, and clinical exposure. For example, students report that participation in end of life care allows for personal growth, shifts in perception, and opportunities to build therapeutic relationships with their clients [44, 47, 48].

Preliminary items generated from the literature resulted in 56 questions from 11 published sources (See Table 3). These items were constructed across four main categories: (i) socio-demographic questions; (ii) end of life care questions; (iii) knowledge about MAiD; or (iv) comfort and willingness to participate in MAiD. Knowledge questions were refined to reflect current MAiD legislation, policies, and regulatory frameworks. Falconer [39] and Freeman [45] studies were foundational sources for item selection. Additionally, four case studies were written to reflect the most recent anticipated changes to MAiD legislation and all used the same open-ended core questions to address respondents’ perspectives about the patient’s right to make the decision, comfort in assisting a physician or NP to administer MAiD in that scenario, and hypothesized comfort about serving as a primary provider if qualified as an NP in future. Response options for the survey were also constructed during this stage and included: open text, categorical, yes/no*,* and Likert scales.

**Table 3:** Origin of items from Phase 1 generation

Survey Questions	Origin of Questions
1. Please enter your age in years	Bendiane et al. (2007); Falconer et. al (2019); Freeman et. al (2020); Hosseinzadeh & Rafiei (2019); Inghelbrecht et. al (2009), Lavoie et. al (2016); Pesut et al. (2020)
2. What sex you were assigned at birth?	Falconer et. al (2019); Inghelbrecht et. al (2009); CIHR (2023)
3. What gender to you identify as?	Bendiane et al. (2007); Freeman et. al (2020); Green et. al (2022); Pesut et al. (2020)
4. Please enter what country you were born in	Falconer et al. (2019)
5. Please enter what province or territory you were born in if you were born in Canada	Falconer et al. (2019)
6. What is your religion or faith expression?	Falconer et al. (2019); Freeman et. al (2020); Green et. al (2022); Lavoie et. al (2016); McMechan, Bruce & Beuthin (2020); Pesut et al. (2020)
7. Religious attendance (days per week)	Falconer et al. (2019);
8. Importance of religion, spirituality or faith	Inghelbrecht et.al., (2009);
9. Have you cared for a patient at end-of-life?	Bendiane et al. (2007); Freeman et. al (2020)
10. Have you cared for a patient at end-of-life in the last 12 months?	Green et. al (2020); Inghelbrecht et. al (2009)
11. Have you discussed end-of-life issues with a patient?	Bendiane et al. (2007); Inghelbrecht et. al (2009)
12. Have you attended or observed a death in the practice setting?	McMechan, Bruce & Beuthin (2019); Lavoie et. al (2016); Falconer et. al (2019)
13. Have you cared for a patient who has requested MAiD?	McMechan, Bruce & Beuthin (2019); Beuthin, Bruce & Scaia (2018)
14. Have you cared for a patient who has received MAiD?	McMechan, Bruce & Beuthin (2019); Beuthin, Bruce & Scaia (2018)
15. Have you been in the room when a patient received MAiD?	McMechan, Bruce & Beuthin (2019); Beuthin, Bruce & Scaia (2018)
16. Did your nursing education include content on MAiD?	Falconer et. al (2019); Ozcelik et. al (2014); McMechan, Bruce & Beuthin (2019)
17. What is your current level of knowledge around the legal responsibilities of the Registered Nurse in MAiD?	Freeman et. al (2020)
18. Do you feel like you have enough information to take part in a discussion about MAiD with other nursing students ?	Freeman et. al (2020)
19. Do you feel like you have enough information to take part in a discussion about MAiD with patients?	Freeman et. al (2020)
20. Are you aware of the federal legislation on MAiD Bill C-14 in Canada?	Falconer et. al (2019), Canadian MAiD legislation
21. Are you aware of the current eligibility criteria for MAiD in Canada?	Freeman et. al (2019), Canadian MAiD legislation
22. Are you aware of the safeguards in place within the MAiD legislation?	Canadian MAiD legislation
23. Are you aware of the recent legislation changes for eligibility criteria (C-7) as of March 2021 for MAiD in Canada?	Freeman et. al (2020); Council of Canadian Academies (2018)
24. I find it easy to discuss MAiD	Freeman et. al (2020)
25. I am in support of Nurse Practitioners providing MAiD	Freeman et. al (2020)
26. I am in support of Physicians providing MAiD	Falconer et. al (2019); Freeman et. al (2020)
27. A person has the right to decide on their own death	Freeman et. al (2020)
28. Patients should have access to palliative care before accessing MAiD	Freeman et. al (2020)
29. My attitude towards MAiD is conflicted	Freeman et. al (2020)
30. I accept MAiD as part of Canadian healthcare	Freeman et. al (2020)
31. My view on MAiD is impacted by my religious or spiritual beliefs	Falconer et. al (2019); Freeman et. al (2020)
32. My undergraduate nursing education has shaped my views on MAiD	McMechan, Bruce & Beuthin (2019)
33. I feel prepared to care for a client requesting MAiD	McMechan, Bruce & Beuthin (2019); Freeman et. al (2020)
34. I feel as though I know what conscientious objection is	Pesut, Thorne & Greig (2019); Canadian MAiD legislation
35. I believe nurses have the right to conscientiously object to participating in MAiD	Pesut, Thorne & Greig (2019), Canadian MAiD legislation
36. I know the steps to follow to declare conscientious objection	Pesut, Thorne & Greig (2019); Canadian MAiD legislation
37. I am comfortable having conversations about MAiD with patients	Freeman et. al (2020)
38. I am willing to start an intravenous (IV) for a patient receiving MAiD	Pesut, Thorne & Greig (2019)
39. I am willing to care for patients and their families during the MAiD process	Freeman et. al (2020)
40. I am comfortable caring for the patients’ body after a MAiD death	Beuthin, Bruce & Scaia (2018)
41. I am comfortable working with families during the bereavement period following a MAiD death	McMechan, Bruce & Beuthin (2019)
42. I am willing to assist the NP or physician to administer a MAiD death	Freeman et. al (2020)
43. I am willing to become a MAiD assessor in my future career	Falconer et. al (2019)
44. I am willing to become a MAiD provider in my future career	Falconer et. al (2019)
45–47. Case study #1: MAiD and a mature minor	Council of Canadian Academies (2018)
48–50. Case study #2: MAiD when psychiatric illness is underlying condition	Council of Canadian Academies (2018); Freeman et. al (2020)
51–53. Case study #3: MAiD when death is not reasonably foreseeable	Council of Canadian Academies (2018)
54–56. Case Study #4: MAiD using an advanced request	Council of Canadian Academies (2018)

### Phase 2: faculty expert panel review

Of the 56 items presented to the faculty panel, 54 questions reached 75% consensus. However, based upon the qualitative responses 9 items were removed largely because they were felt to be repetitive. Items that generated the most controversy were related to measuring religion and spirituality in the Canadian context, defining end of life care when there is no agreed upon time frames (e.g., last days, months, or years), and predicting willingness to be involved in a future events – thus predicting their future selves. Phase 2, round 1 resulted in an initial set of 47 items which were then presented back to the faculty panel in round 2.

**Fig. 2:**
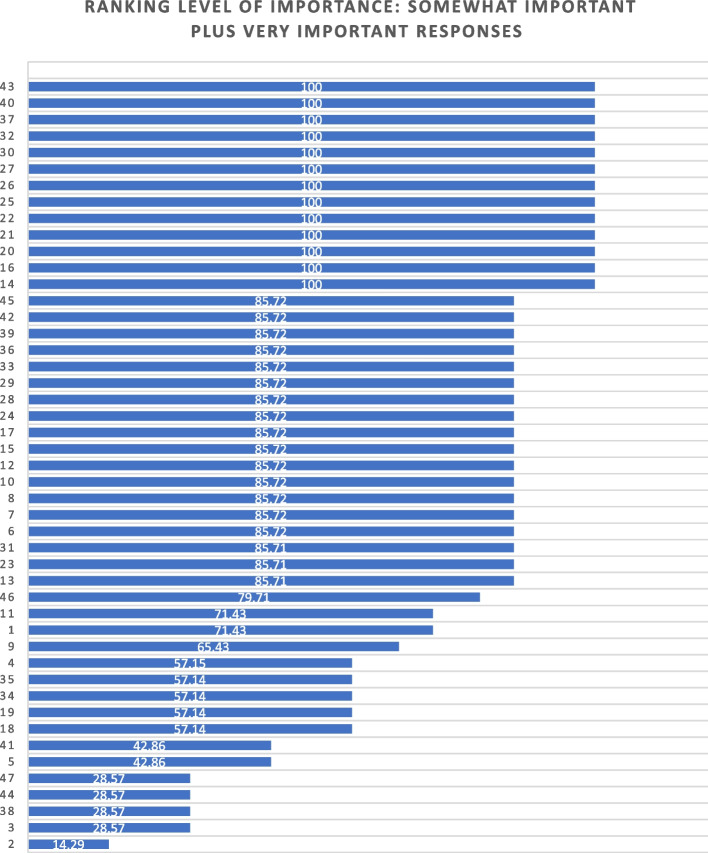
Ranking level of importance for survey items

Of the 47 initial questions presented to the panel in round 2, 45 reached a level of consensus of 75% or greater, and 34 of these questions reached a level of 100% consensus [27] of which all participants chose to include without any adaptations) For each question, level of importance was determined based on a 5-point Likert scale (1 = very unimportant, 2 = somewhat unimportant, 3 = neutral, 4 = somewhat important, and 5 = very important). Figure 2 provides an overview of the level of importance assigned to each item.

After round 2, a careful analysis of participant comments and level of importance was completed by the research team. While the main method of survey item development came from participants’ response to the first round of Delphi consensus ratings, level of importance was used to assist in the decision of whether to keep or modify questions that created controversy, or that rated lower in the include/exclude/adapt portion of the Delphi. Survey items that rated low in level of importance included questions about future roles, sex and gender, and religion/spirituality. After deliberation by the research committee, these questions were retained in the survey based upon the importance of these variables in the scientific literature.

Of the 47 questions remaining from Phase 2, round 2, four were revised. In addition, the two questions that did not meet the 75% cut off level for consensus were reviewed by the research team. The first question reviewed was *What is your comfort level with providing a MAiD death in the future if you were a qualified NP*? Based on a review of participant comments, it was decided to retain this question for the cognitive interviews with students in the final phase of testing. The second question asked about impacts on respondents’ views of MAiD and was changed from one item with 4 subcategories into 4 separate items, resulting in a final total of 51 items for phase 3. The revised survey was then brought forward to the cognitive interviews with student participants in Phase 3. (see Supplementary Material 1 for a complete description of item modification during round 2).

### Phase 3. Outcomes of cognitive interview focus group

Of the 51 items reviewed by student participants, 29 were identified as clear with little or no discussion. Participant comments for the remaining 22 questions were noted and verified against the audio recording. Following content analysis of the comments, four key themes emerged through the student discussion: unclear or ambiguous wording; difficult to answer questions; need for additional response options; and emotional response evoked by questions. An example of unclear or ambiguous wording was a request for clarity in the use of the word “sufficient” in the context of assessing an item that read “My nursing education has provided *sufficient* content about the nursing role in MAiD.” “Sufficient” was viewed as subjective and “laden with…complexity that distracted me from the question.” The group recommended rewording the item to read “My nursing education has provided enough content for me to care for a patient considering or requesting MAiD.”

An example of having difficulty answering questions related to limited knowledge related to terms used in the legislation such as such as *safeguards*, *mature minor*, *eligibility criteria*, and *conscientious objection.* Students were unclear about what these words meant relative to the legislation and indicated that this lack of clarity would hamper appropriate responses to the survey. To ensure that respondents are able to answer relevant questions, student participants recommended that the final survey include explanation of key terms such as *mature minor* and *conscientious objection* and an overview of current legislation.

Response options were also a point of discussion. Participants noted a lack of distinction between response options of *unsure* and *unable to say*. Additionally, scaling of attitudes was noted as important since perspectives about MAiD are dynamic and not dichotomous “agree or disagree” responses. Although the faculty expert panel recommended the integration of the demographic variables of religious and/or spiritual remain as a single item, the student group stated a preference to have religion and spirituality appear as separate items. The student focus group also took issue with separate items for the variables of sex and gender, specifically that non-binary respondents might feel othered or “outed” particularly when asked to identify their sex. These variables had been created based upon best practices in health research but students did not feel they were appropriate in this context [49]. Finally, students agreed with the faculty expert panel in terms of the complexity of projecting their future involvement as a Nurse Practitioner. One participant stated: “I certainly had to like, whoa, whoa, whoa. Now let me finish this degree first, please.” Another stated, “I'm still imagining myself, my future career as an RN.”

Finally, student participants acknowledged the array of emotions that some of the items produced for them. For example, one student described positive feelings when interacting with the survey. “Brought me a little bit of feeling of joy. Like it reminded me that this is the last piece of independence that people grab on to.” Another participant, described the freedom that the idea of an advance request gave her. “The advance request gives the most comfort for me, just with early onset Alzheimer’s and knowing what it can do.” But other participants described less positive feelings. For example, the mature minor case study yielded a comment: “This whole scenario just made my heart hurt with the idea of a child requesting that.”

Based on the data gathered from the cognitive interview focus group of nursing students, revisions were made to 11 closed-ended questions (see Table 4) and 3 items were excluded. In the four case studies, the open-ended question related to a respondents’ hypothesized actions in a future role as NP were removed. The final survey consists of 45 items including 4 case studies (see Supplementary Material 3).

**Table 4:** Questions adapted or removed based on phase 3 cognitive interview focus group

Questions	Summary of participant comments	Decision
2.What sex were you assigned at birth?	Triggering, confusing, questioning relevance to survey	Exclude
3.What gender do you identify as?	Felt “othered”, word other was a “problem”, feelings of frustration	Revised: a) Male, b) Non-binary, c) Female, 4) Prefer to self-describe
6.Do you consider yourself religious and/or spiritual?	Subjective, relationship to question is often “in flux”, recommend separating the two	Excluded
7.How important is religion and/or spirituality to you?	Spirituality and being spiritual might be different, “had to pause”, recommend separating	Revised: Split into two separate questions
9.Have you discussed or been in discussions with a patient regarding end-of-life issues?	Unclear if “being involved” meant having or witnessing discussions	Revised: Have you discussed or observed discussions with a patient regarding end-of-life issues
10.Have you cared for a patient who has requested MAiD?	Initial request or who has been approved? Is this patient actively considering? Have they gone through legal paper work?	Revised: Have you cared for a patient who was actively considering MAiD?
11.Have you cared for a patient who later received MAiD?	Unsure if clients they worked with actually received MAiD. More often this is unknown for students as work with patients in short rotations	Revised: added “unsure” response
13.My nursing education has provided sufficient content about the nursing role in MAiD	Unclear on word sufficient can be subjective/ambiguous. Unsure what ‘enough’ content would be. Wondering if this is about sufficient content or availability of content	Revised: My nursing education has provided enough content for me to care for a patient considering or receiving MAiD. Add “unsure” option
15.I have enough information to take part in a discussion with other nursing students about MAiD	Clarity on word discussion, does this mean having accurate/meaningful discussions? Is this in relation to specific information about MAiD?	Revised: I have enough understanding about MAiD to take part in a meaningful discussion with other nursing students
24.My attitude towards MAiD is conflicted	Should be first question in this section as it invites student to reflect on how they situate themselves and how they engage in the process	Moved to question #21 in survey
25. I accept MAiD as part of Canadian healthcare	Brought up feelings and emotions that this is not a question asked of other areas in nursing	Revised: I believe MAiD should be a part of the Canadian healthcare system
31. My view of MAiD is strongly impacted but my personal and/or professional experiences	Separate these two. Personal may impact someone different than professional	Revised: Split into two separate questions
38. I am willing to become a MAiD assessor in my future career (if I was an NP)	Did not know meaning of assessor, difficult to answer question into the future. Career decisions change rapidly in nursing so only captures one point in time	Exclude
39.I am willing to become a MAID provider I my future career (if I was NP)	Ambiguous	Exclude

## Discussion

The aim of this study was to develop and validate a survey that can be used to track the growth of knowledge about MAiD among nursing students over time, inform training programs about curricular needs, and evaluate attitudes and willingness to participate in MAiD at time-points during training or across nursing programs over time.

The faculty expert panel and student participants in the cognitive interview focus group identified a need to establish core knowledge of the terminology and legislative rules related to MAiD. For example, within the cognitive interview group of student participants, several acknowledged lack of clear understanding of specific terms such as “conscientious objector” and “safeguards.” Participants acknowledged discomfort with the uncertainty of not knowing and their inclination to look up these terms to assist with answering the questions. This survey can be administered to nursing or pre-nursing students at any phase of their training within a program or across training programs. However, in doing so it is important to acknowledge that their baseline knowledge of MAiD will vary. A response option of “not sure” is important and provides a means for respondents to convey uncertainty. If this survey is used to inform curricular needs, respondents should be given explicit instructions not to conduct online searches to inform their responses, but rather to provide an honest appraisal of their current knowledge and these instructions are included in the survey (see Supplementary Material 3).

Some provincial regulatory bodies have established core competencies for entry-level nurses that include MAiD. For example, the BC College of Nurses and Midwives (BCCNM) requires “knowledge about ethical, legal, and regulatory implications of medical assistance in dying (MAiD) when providing nursing care.” (10 p. 6) However, across Canada curricular content and coverage related to end of life care and MAiD is variable [23]. Given the dynamic nature of the legislation that includes portions of the law that are embargoed until 2024, it is important to ensure that respondents are guided by current and accurate information. As the law changes, nursing curricula, and public attitudes continue to evolve, inclusion of core knowledge and content is essential and relevant for investigators to be able to interpret the portions of the survey focused on attitudes and beliefs about MAiD. Content knowledge portions of the survey may need to be modified over time as legislation and training change and to meet the specific purposes of the investigator.

Given the sensitive nature of the topic, it is strongly recommended that surveys be conducted anonymously and that students be provided with an opportunity to discuss their responses to the survey. A majority of feedback from both the expert panel of faculty and from student participants related to the wording and inclusion of demographic variables, in particular religion, religiosity, gender identity, and sex assigned at birth. These and other demographic variables have the potential to be highly identifying in small samples. In any instance in which the survey could be expected to yield demographic group sizes less than 5, users should eliminate the demographic variables from the survey. For example, the profession of nursing is highly dominated by females with over 90% of nurses who identify as female [50]. Thus, a survey within a single class of students or even across classes in a single institution is likely to yield a small number of male respondents and/or respondents who report a difference between sex assigned at birth and gender identity. When variables that serve to identify respondents are included, respondents are less likely to complete or submit the survey, to obscure their responses so as not to be identifiable, or to be influenced by social desirability bias in their responses rather than to convey their attitudes accurately [51]. Further, small samples do not allow for conclusive analyses or interpretation of apparent group differences. Although these variables are often included in surveys, such demographics should be included only when anonymity can be sustained. In small and/or known samples, highly identifying variables should be omitted.

There are several limitations associated with the development of this survey. The expert panel was comprised of faculty who teach nursing students and are knowledgeable about MAiD and curricular content, however none identified as a conscientious objector to MAiD. Ideally, our expert panel would have included one or more conscientious objectors to MAiD to provide a broader perspective. Review by practitioners who participate in MAiD, those who are neutral or undecided, and practitioners who are conscientious objectors would ensure broad applicability of the survey. This study included one student cognitive interview focus group with 5 self-selected participants. All student participants had held discussions about end of life care with at least one patient, 4 of 5 participants had worked with a patient who requested MAiD, and one had been present for a MAiD death. It is not clear that these participants are representative of nursing students demographically or by experience with end of life care. It is possible that the students who elected to participate hold perspectives and reflections on patient care and MAiD that differ from students with little or no exposure to end of life care and/or MAiD. However, previous studies find that most nursing students have been involved with end of life care including meaningful discussions about patients’ preferences and care needs during their education [40, 44, 47, 48, 52]. Data collection with additional student focus groups with students early in their training and drawn from other training contexts would contribute to further validation of survey items.

Future studies should incorporate pilot testing with small sample of nursing students followed by a larger cross-program sample to allow evaluation of the psychometric properties of specific items and further refinement of the survey tool. Consistent with literature about the importance of leadership in the context of MAiD [12, 53, 54], a study of faculty knowledge, beliefs, and attitudes toward MAiD would provide context for understanding student perspectives within and across programs. Additional research is also needed to understand the timing and content coverage of MAiD across Canadian nurse training programs’ curricula.

## Conclusion

The implementation of MAiD is complex and requires understanding of the perspectives of multiple stakeholders. Within the field of nursing this includes clinical providers, educators, and students who will deliver clinical care. A survey to assess nursing students’ attitudes toward and willingness to participate in MAiD in the Canadian context is timely, due to the legislation enacted in 2016 and subsequent modifications to the law in 2021 with portions of the law to be enacted in 2027. Further development of this survey could be undertaken to allow for use in settings with practicing nurses or to allow longitudinal follow up with students as they enter practice. As the Canadian landscape changes, ongoing assessment of the perspectives and needs of health professionals and students in the health professions is needed to inform policy makers, leaders in practice, curricular needs, and to monitor changes in attitudes and practice patterns over time.

### Supplementary Information

Supplementary Material 1.

Supplementary Material 2.

Supplementary Material 3.

## Data Availability

The datasets used and/or analysed during the current study are not publicly available due to small sample sizes, but are available from the corresponding author on reasonable request.

## Abbreviations

BCCNM: British Columbia College of Nurses and Midwives
MAiD: Medical assistance in dying
NP: Nurse practitioner
RN: Registered nurse
UBCO: University of British Columbia Okanagan

## References

[1] Nicol J, Tiedemann M. Legislative Summary: Bill C-14: An Act to amend the Criminal Code and to make related amendments to other Acts (medical assistance in dying). Available from: https://lop.parl.ca/staticfiles/PublicWebsite/Home/ResearchPublications/LegislativeSummaries/PDF/42-1/c14-e.pdf.

[2] Downie J, Scallion K. Foreseeably unclear. The meaning of the “reasonably foreseeable” criterion for access to medical assistance in dying in Canada. Dalhousie Law J. 2018;41(1):23–57.

[3] Nicol J, Tiedeman M (2021). Legislative summary of Bill C-7: an act to amend the criminal code (medical assistance in dying).

[4] Council of Canadian Academies. The state of knowledge on medical assistance in dying where a mental disorder is the sole underlying medical condition. Ottawa; 2018. Available from: https://cca-reports.ca/wp-content/uploads/2018/12/The-State-of-Knowledge-on-Medical-Assistance-in-Dying-Where-a-Mental-Disorder-is-the-Sole-Underlying-Medical-Condition.pdf.

[5] Council of Canadian Academies. The state of knowledge on advance requests for medical assistance in dying. Ottawa; 2018. Available from: https://cca-reports.ca/wp-content/uploads/2019/02/The-State-of-Knowledge-on-Advance-Requests-for-Medical-Assistance-in-Dying.pdf.

[6] Council of Canadian Academies. The state of knowledge on medical assistance in dying for mature minors. Ottawa; 2018. Available from: https://cca-reports.ca/wp-content/uploads/2018/12/The-State-of-Knowledge-on-Medical-Assistance-in-Dying-for-Mature-Minors.pdf.

[7] Health Canada. Third annual report on medical assistance in dying in Canada 2021. Ottawa; 2022. [cited 2023 Oct 23]. Available from: https://www.canada.ca/en/health-canada/services/medical-assistance-dying/annual-report-2021.html.

[8] Banner D, Schiller CJ, Freeman S (2019). Medical assistance in dying: a political issue for nurses and nursing in Canada. Nurs Philos.

[9] Pesut B, Thorne S, Stager ML, Schiller CJ, Penney C, Hoffman C (2019). Medical assistance in dying: a review of Canadian nursing regulatory documents. Policy Polit Nurs Pract.

[10] College of Registered Nurses of British Columbia. Scope of practice for registered nurses [Internet]. Vancouver; 2018. Available from: https://www.bccnm.ca/Documents/standards_practice/rn/RN_ScopeofPractice.pdf.

[11] Pesut B, Thorne S, Schiller C, Greig M, Roussel J, Tishelman C (2020). Constructing good nursing practice for medical assistance in dying in Canada: an interpretive descriptive study. Global Qual Nurs Res.

[12] Pesut B, Thorne S, Schiller CJ, Greig M, Roussel J (2020). The rocks and hard places of MAiD: a qualitative study of nursing practice in the context of legislated assisted death. BMC Nurs.

[13] Pesut B, Greig M, Thorne S, Burgess M, Storch JL, Tishelman C (2020). Nursing and euthanasia: a narrative review of the nursing ethics literature. Nurs Ethics.

[14] Pesut B, Thorne S, Storch J, Chambaere K, Greig M, Burgess M (2020). Riding an elephant: a qualitative study of nurses' moral journeys in the context of Medical Assistance in Dying (MAiD). Journal Clin Nurs.

[15] Lamb C, Babenko-Mould Y, Evans M, Wong CA, Kirkwood KW (2018). Conscientious objection and nurses: results of an interpretive phenomenological study. Nurs Ethics.

[16] Wright DK, Chan LS, Fishman JR, Macdonald ME (2021). "Reflection and soul searching:" Negotiating nursing identity at the fault lines of palliative care and medical assistance in dying. Social Sci & Med.

[17] Beuthin R, Bruce A, Scaia M (2018). Medical assistance in dying (MAiD): Canadian nurses’ experiences. Nurs Forum.

[18] Bruce A, Beuthin R. Medically assisted dying in Canada: "Beautiful Death" is transforming nurses' experiences of suffering. The Canadian J Nurs Res | Revue Canadienne de Recherche en Sci Infirmieres. 2020;52(4):268–77. 10.1177/0844562119856234. 10.1177/0844562119856234 31188639

[19] Canadian Nurses Association. Code of ethics for registered nurses. Ottawa; 2017. Available from: https://www.cna-aiic.ca/en/nursing/regulated-nursing-in-canada/nursing-ethics.

[20] Canadian Nurses Association. National nursing framework on Medical Assistance in Dying in Canada. Ottawa: 2017. Available from: https://www.virtualhospice.ca/Assets/cna-national-nursing-framework-on-maidEng_20170216155827.pdf.

[21] Pesut B, Thorne S, Greig M (2020). Shades of gray: conscientious objection in medical assistance in dying. Nursing Inq.

[22] Durojaiye A, Ryan R, Doody O (2023). Student nurse education and preparation for palliative care: a scoping review. PLoS ONE.

[23] McMechan C, Bruce A, Beuthin R. Canadian nursing students’ experiences with medical assistance in dying | Les expériences d’étudiantes en sciences infirmières au regard de l’aide médicale à mourir. Qual Adv Nurs Educ - Avancées en Formation Infirmière. 2019;5(1). 10.17483/2368-6669.1179.

[24] Adler M, Ziglio E. Gazing into the oracle. The Delphi method and its application to social policy and public health. London: Jessica Kingsley Publishers; 1996

[25] Keeney S, Hasson F, McKenna H (2006). Consulting the oracle: ten lessons from using the Delphi technique in nursing research. J Adv Nurs.

[26] Keeney S, Hasson F, McKenna H. The Delphi technique in nursing and health research. 1st ed. City: Wiley; 2011.

[27] Willis GB. Cognitive interviewing: a tool for improving questionnaire design. 1st ed. Thousand Oaks, Calif: Sage; 2005. ISBN: 9780761928041

[28] Lamb C, Evans M, Babenko-Mould Y, Wong CA, Kirkwood EW (2017). Conscience, conscientious objection, and nursing: a concept analysis. Nurs Ethics.

[29] Lamb C, Evans M, Babenko-Mould Y, Wong CA, Kirkwood K (2018). Nurses’ use of conscientious objection and the implications of conscience. J Adv Nurs.

[30] de Vaus D (2014). Surveys in social research.

[31] Boateng GO, Neilands TB, Frongillo EA, Melgar-Quiñonez HR, Young SL (2018). Best practices for developing and validating scales for health, social, and behavioral research: A primer. Front Public Health.

[32] Puchta C, Potter J (2004). Focus group practice.

[33] Streiner DL, Norman GR, Cairney J (2015). Health measurement scales: a practical guide to their development and use.

[34] Hsieh H-F, Shannon SE (2005). Three approaches to qualitative content analysis. Qual Health Res.

[35] Adesina O, DeBellis A, Zannettino L (2014). Third-year Australian nursing students' attitudes, experiences, knowledge, and education concerning end-of-life care. Int J of Palliative Nurs.

[36] Bator EX, Philpott B, Costa AP (2017). This moral coil: a cross-sectional survey of Canadian medical student attitudes toward medical assistance in dying. BMC Med Ethics.

[37] Beuthin R, Bruce A, Scaia M (2018). Medical assistance in dying (MAiD): Canadian nurses’ experiences. Nurs Forum.

[38] Brown J, Goodridge D, Thorpe L, Crizzle A. What is right for me, is not necessarily right for you: the endogenous factors influencing nonparticipation in medical assistance in dying. Qual Health Res. 2021;31(10):1786–1800. 10.1177/10497323211008843 PMC8446887 33938306

[39] Falconer J, Couture F, Demir KK, Lang M, Shefman Z, Woo M (2019). Perceptions and intentions toward medical assistance in dying among Canadian medical students. BMC Med Ethics.

[40] Green G, Reicher S, Herman M, Raspaolo A, Spero T, Blau A (2022). Attitudes toward euthanasia—dual view: Nursing students and nurses. Death Stud.

[41] Hosseinzadeh K, Rafiei H (2019). Nursing student attitudes toward euthanasia: a cross-sectional study. Nurs Ethics.

[42] Ozcelik H, Tekir O, Samancioglu S, Fadiloglu C, Ozkara E (2014). Nursing students' approaches toward euthanasia. Omega (Westport).

[43] Canning SE, Drew C. Canadian nursing students’ understanding, and comfort levels related to medical assistance in dying. Qual Adv Nurs Educ - Avancées en Formation Infirmière. 2022;8(2). 10.17483/2368-6669.1326.

[44] Edo-Gual M, Tomás-Sábado J, Bardallo-Porras D, Monforte-Royo C (2014). The impact of death and dying on nursing students: an explanatory model. J Clin Nurs.

[45] Freeman LA, Pfaff KA, Kopchek L, Liebman J (2020). Investigating palliative care nurse attitudes towards medical assistance in dying: an exploratory cross-sectional study. J Adv Nurs.

[46] Brown J, Goodridge D, Thorpe L, Crizzle A (2021). “I am okay with it, but I am not going to do it:” the exogenous factors influencing non-participation in medical assistance in dying. Qual Health Res.

[47] Dimoula M, Kotronoulas G, Katsaragakis S, Christou M, Sgourou S, Patiraki E (2019). Undergraduate nursing students' knowledge about palliative care and attitudes towards end-of-life care: A three-cohort, cross-sectional survey. Nurs Educ Today.

[48] Matchim Y, Raetong P (2018). Thai nursing students' experiences of caring for patients at the end of life: a phenomenological study. Int J Palliative Nurs.

[49] Canadian Institute for Health Research. Sex and gender in health research [Internet]. Ottawa: CIHR; 2021 [cited 2023 Oct 23]. Available from: https://cihr-irsc.gc.ca/e/50833.html.

[50] Canadian Nurses’ Association. Nursing statistics. Ottawa: CNA; 2023 [cited 2023 Oct 23]. Available from: https://www.cna-aiic.ca/en/nursing/regulated-nursing-in-canada/nursing-statistics.

[51] Krumpal I (2013). Determinants of social desirability bias in sensitive surveys: a literature review. Qual Quant.

[52] Ferri P, Di Lorenzo R, Stifani S, Morotti E, Vagnini M, Jiménez Herrera MF (2021). Nursing student attitudes toward dying patient care: a European multicenter cross-sectional study. Acta Bio Medica Atenei Parmensis.

[53] Beuthin R, Bruce A (2018). Medical assistance in dying (MAiD): Ten things leaders need to know. Nurs Leadership.

[54] Thiele T, Dunsford J (2019). Nurse leaders' role in medical assistance in dying: a relational ethics approach. Nurs Ethics.

