# Supplementary material for: Developing a survey to measure nursing students’ knowledge, attitudes and beliefs, influences, and willingness to be involved in Medical Assistance in Dying (MAiD): a mixed method modified e-Delphi study
Source: BMC Nurs. 2024 May 14;23:326. doi: 10.1186/s12912-024-01984-z (PMC11092000; doi:10.1186/s12912-024-01984-z)
Supplement: Supplementary file 1 — Supplementary Material 1. [file 12912_2024_1984_MOESM1_ESM.docx]

Additional File 1

*Decision for Survey Questions: Delphi Round Two*

| Question | Consensus Level (%) | Participant Comments | Decision |
| --- | --- | --- | --- |
| 1. Please enter your age in years | 100 | It is possible some students may be younger than 20 | Revised: Included category for less than 20 |
| 2.What sex you were assigned at birth? | 75 |  | No change |
| 3.What gender to you identify as? | 75 | These are not gender categories. Here are possible options: Woman.  Man.  Transgender.  Non-binary/non-conforming.  Prefer not to respond. | No change |
| 4. Please enter what country you were primarily raised in? | 87.5 |  | No change |
| 5. Please enter what province or territory you were born in if you were born in Canada | 75 | If a person was not born in Canada - how will they respond? | No change: Question will only show for those who enter Canada |
| 6. Do you consider yourself religious and/or spiritual? | 100 | It seems the terms are important to identify or explain here religious and spiritual are not necessarily very different for some | No change |
| 7. Importance of religion or faith | 100 |  | Revised: How important is religion and/or spirituality to you? |
| 8. Have you cared for a patient who has died during the period of time you were looking after them? | 100 |  | No change |
| 9. Have you discussed or been involved in discussions with a patient regarding end-of-life issues? | 100 |  | No change |
| 10. Have you cared for a patient who has requested MAiD? | 100 |  | No change |
| 11. Have you cared for a patient who has received MAiD? | 100 |  | No change |
| 12. Have you been in the room when a patient received MAiD? | 100 |  | No change |
| 13. My nursing education has provided sufficient content about the nursing role in MAiD | 100 |  | No change |
| 14. Please rate your understanding of the role of the Registered Nurse in MAiD | 100 |  | No change |
| 15. I feel like I have enough information to take part in a discussion about MAiD with other nursing students | 100 |  | No change |
| 16. I know the current eligibility criteria for MAiD in Canada | 100 |  | No change |
| 17. I know the safeguards in place within the MAiD legislation? | 100 |  | No change |
| 18. I am in support of Nurse Practitioners providing MAiD | 87.5 |  | No change |
| 19. I am in support of Physicians providing MAiD | 87.5 |  | No change |
| 20. A person has the right to decide on the timing of their own death | 100 |  | No change |
| 21. My attitude towards MAiD is conflicted | 100 |  | No change |
| 22. I accept MAiD as part of Canadian healthcare | 100 |  | No change |
| 23. My view on MAiD is impacted by my  a) religious or spiritual beliefs  b) family  c) co-workers  d) friends | 100 | Could include personal/professional experiences as an option  I am thinking we are missing out a strong influence of one’s past experiences other than people and spiritual beliefs. I would like to see an option E for lived experiences | Revised: Include personal and/or professional experiences as sub-category |
| 24. My undergraduate nursing education has shaped my views on MAiD | 100 | Has helped to shape or "helped to inform" | Revised: My undergraduate nursing education has helped shape my views on MAiD |
| 25. I feel prepared to care for a client requesting MAiD | 100 |  | No change |
| 26. I understand what is meant by conscientious objection | 100 |  | No change |
| 27. I believe nurses have the right to conscientiously object to participating in MAiD | 100 |  | No change |
| 28. I know the steps to follow to declare conscientious objection | 100 |  | No change |
| 29. As an RN I would be willing to start an intravenous (IV) for a patient receiving MAiD | 100 |  | No change |
| 30. I am willing to care for patients and their families during the MAiD process within my scope of practice | 100 |  | No change |
| 31. I am comfortable caring for the patients’ body after a MAiD death | 87.5 |  | No change |
| 32. I am comfortable working with families during the bereavement period following a MAiD death | 100 |  | No change |
| 33. I am willing to assist the NP or physician to administer a MAiD death within my scope of practice | 100 |  | No change |
| 34. I am willing to become a MAiD assessor in my future career | 87.5 |  | No change |
| 35. I am willing to become a MAiD provider in my future career | 87.5 |  | No change |
| Case study #1: MAiD and a mature minor  36. What is your comfort level with a mature minor’s right to make this decision? | 100 |  | No Change |
| 37. What is your comfort level with assisting the physician/NP to administer death? | 100 |  | No change |
| 38. What is your comfort level with providing a MAiD death in the future if you were a qualified NP? | 75 | Maybe difficult to predict future comfort level? very few likely to be qualified as NPs | No change |
| Case study #2: MAiD when psychiatric illness is underlying condition  39. | 100 |  | No change |
| 40. What is your comfort level with assisting the physician/NP to administer death? | 100 |  | No change |
| 41. What is your comfort level with providing a MAiD death in the future if you were a qualified NP? | 75 |  | No change |
| Case study #3: MAiD when death is not reasonably foreseeable  42. What is your comfort level with the patients right to make this decision? | 100 |  | No change |
| 43.What is your comfort level with assisting the physician/NP to administer death? | 100 |  | No change |
| 44. What is your comfort level with providing a MAiD death in the future if you were a qualified NP? | 62.5 |  | No change: Experts in committee agreed to test this question in the focus group with students. |
| Case Study #4: MAiD using an advanced request  45. What is your comfort level with the patients right to make this decision? | 100 |  | No change |
| 46. What is your comfort level with assisting the physician/NP to administer death? | 100 |  |  |
| 47. What is your comfort level with providing a MAiD death in the future if you were a qualified NP? | 62.5 |  | No change: Experts in the committee agreed to test this question in the focus group with students. |
