# Supplementary material for: Developing a survey to measure nursing students’ knowledge, attitudes and beliefs, influences, and willingness to be involved in Medical Assistance in Dying (MAiD): a mixed method modified e-Delphi study
Source: BMC Nurs. 2024 May 14;23:326. doi: 10.1186/s12912-024-01984-z (PMC11092000; doi:10.1186/s12912-024-01984-z)
Supplement: Supplementary file 2 — Supplementary Material 2. [file 12912_2024_1984_MOESM2_ESM.docx]

# Interview Guide for Focus Group

**Cognitive Interview Framework:** Semi-scripted

**Interview Timing:** Approximately 60-90 minutes

**Introduction: Review consent form**

**Explanation of the purpose of the study: “**The purpose of this study to develop and initially validate a survey that can be utilized at a future date to examine nursing students’ attitudes toward, and willingness to participate in, Medical Assistance in Dying (MAID) in the Canadian context. Currently there are no known quantitative studies with nursing students in the Canadian context, and there are no existing surveys that could be adapted for the purpose of this future research. During this interview the goal is for you to review questions and comment on the clarity and wording of the questions.”

**Audio Recording of Interview: “**Please note this focused group interview will be audio recorded, and through the interview I will also be taking notes.” Request permission to record session.

**Preparing and training for the ‘think aloud’ technique:**

“Thinking aloud is talking through your thought process out loud to the group. The think aloud technique can be used to capture your understanding of each survey question and help you articulate its clarity. There are no wrong answers, I am interested in knowing what you are thinking”

Sample practice question:

*“Try to visualize a place where you’ve lived. It could be your current home or a place you’ve lived in the past. As you think about that place, think about how many windows there are in that place or in part of that place if it’s very large. As you move through the place and count the windows, tell me what you are seeing and thinking about.” (Willis, 1994)*

**Probe examples that may be used following questions:**

“Following the think aloud technique, you may be asked verbal probe questions to further explore your answer and understanding of the questions. Examples of verbal probes that may be used during the interview include”:

| 1. Can you repeat the question in your own words? |
| --- |
| 1. Can you tell me in your own words what this question is asking? |
| 1. Can you tell me what you were thinking when you read this question? |
| 1. What does the word (x) mean to you when its used in this question? |
| 1. How well does this question apply to you? |
| 1. Can you tell me more about your opinion on that? |
| 1. How did you arrive to this answer? |
| 1. Was this easy or difficult to answer? |
| 1. Can you think of any other reasons? |
| 1. How much have you thought about (x)? |
| 1. Is it OK to talk about this survey question or is it uncomfortable? |
| 1. The question used the word (X), does that sound OK to you? Or would you choose something different? |

(If students begin to discuss personal views about MAiD, the interviewer will provide a reminder to avoid discussing personal views and return to the discussion about the survey by moving on to the next question or re stating the previous question.)

**Question period before beginning:**

“Are there any questions about the consent, the ‘think aloud’, or verbal probing techniques before we begin the interview?”

“We will now get started with the cognitive interview”

**Instructions:**

1. Please open the attached file the contains the survey questions.
2. Please read the survey instructions carefully and we will discuss any questions you have about wording or any areas that may be unclear before moving to question #1.
3. Read through the first interview question aloud to yourself (on mute) and then use the ‘think aloud’ technique to describe the question clarity within the group with microphone. When you are ready to think aloud, please use the ‘raise hand’ function on Zoom.
4. You will now repeat this think aloud technique as a group after reading each question

**Closing Discussion and Questions:**

“Thank you very much for your participation in this interview. Please feel free to share any final comments you have, or anything additional you would like to add”
