# Supplementary material for: Developing a survey to measure nursing students’ knowledge, attitudes and beliefs, influences, and willingness to be involved in Medical Assistance in Dying (MAiD): a mixed method modified e-Delphi study
Source: BMC Nurs. 2024 May 14;23:326. doi: 10.1186/s12912-024-01984-z (PMC11092000; doi:10.1186/s12912-024-01984-z)
Supplement: Supplementary file 3 — Supplementary Material 3. [file 12912_2024_1984_MOESM3_ESM.docx]

Table 4 Finalized survey items

Please review the instructions prior to completing this survey.

We understand that legislation and practice of MAiD is quite new and some things will be unfamiliar to you as you answer survey questions. All response options include “Unsure” or “I don’t know” as an option. It’s ok if you don’t know an answer. One purpose of the survey is to inform us about gaps in knowledge and the instructional needs of nursing students. To help inform us about what you know, please do not use outside web resources to answer this survey.

**Part 1. Clinical experience**

| During your training, have you… | Yes | No | Unsure |
| --- | --- | --- | --- |
| Provided clinical care for a patient who has died during the period of time you were looking after them? |  |  |  |
| Discussed or observed discussions with a patient about end of life care issues? |  |  |  |
| Cared for a patient who was actively considering MAiD? |  |  |  |
| Cared for a patient who later received MAiD? |  |  |  |
| Been in the room when a patient received MAiD? |  |  |  |

**Part 2. Education and Preparation**

|  | SA | A | N | D | SD | U |
| --- | --- | --- | --- | --- | --- | --- |
| My nursing education provided me with enough content to care for a patient considering or receiving MAiD |  |  |  |  |  |  |
| I understand the role of the Registered Nurse in MAiD |  |  |  |  |  |  |
| I have enough understanding about MAiD to take part in a meaningful discussion with other nursing students |  |  |  |  |  |  |
| I know the eligibility criteria in place within the current MAiD legislation |  |  |  |  |  |  |
| I know the current safeguards in place within the MAiD legislation |  |  |  |  |  |  |
| I feel prepared to care for a client requesting MAiD |  |  |  |  |  |  |
| I understand what is meant by conscientious objection |  |  |  |  |  |  |

Strongly Agree (SA), Agree (A), Neutral (N), Disagree (D), Strongly Disagree (SD), Unsure (U)

**Part 3. Attitudes and Beliefs**

| My personally held view is… | SA | A | N | D | SD | U |
| --- | --- | --- | --- | --- | --- | --- |
| A person has the right to decide on the timing of their own death |  |  |  |  |  |  |
| MAiD should be part of the Canadian healthcare system |  |  |  |  |  |  |
| Nurses have the right to choose not to participate in MAiD |  |  |  |  |  |  |
| My attitude toward MAiD is conflicted |  |  |  |  |  |  |
| I support Nurse Practitioners providing MAiD |  |  |  |  |  |  |
| I support physicians providing MAiD |  |  |  |  |  |  |

**Part 4. Influences**

| My view about MAiD is influenced by my… | SA | A | N | D | SD | U |
| --- | --- | --- | --- | --- | --- | --- |
| Family |  |  |  |  |  |  |
| Coworkers |  |  |  |  |  |  |
| Friends |  |  |  |  |  |  |
| Personal experiences |  |  |  |  |  |  |
| Religious or spiritual beliefs |  |  |  |  |  |  |
| Professional experiences |  |  |  |  |  |  |
| Undergraduate nursing education |  |  |  |  |  |  |

**Part 5. Future Involvement**

| After I graduate and can work as a registered nurse, I would be willing to… | SA | A | N | D | SD | U |
| --- | --- | --- | --- | --- | --- | --- |
| Start an intravenous (IV) for a patient receiving MAiD |  |  |  |  |  |  |
| Care for patients and their families during the MAiD process within my scope of practice |  |  |  |  |  |  |
| Care for a patient’s body after a MAiD death |  |  |  |  |  |  |
| Care for families during the bereavement period following a MAiD death |  |  |  |  |  |  |
| Assist a NP or physician to administer a MAiD death within my scope of practice |  |  |  |  |  |  |

# Part 6: Clinical Scenarios

MAiD is now available to those whose natural death is not reasonably foreseeable (in other words they are not at end of life). Further, there are several populations who are currently under consideration of becoming eligible to request MAiD: mature minors (adolescents under the age of 18 who can give legal consent, who have demonstrated decision making capacity, and who understand the consequences of medical treatment), those with psychiatric illness as the primary underlying condition, and those desiring to complete an advanced request for MAiD. We are interested in your comfort with MAiD under these special considerations.

**Case Study 1: MAiD when death is not reasonably foreseeable**

You are working in home care, and a 48 year old client with Multiple Sclerosis (MS) makes a written request to their physician for MAiD. The client reports increasing pain and a significant decrease in quality of life in the last year. Currently their death is predicted to be years in the future.

| What is your comfort level with… | VU | SU | N | SC | VC | U |
| --- | --- | --- | --- | --- | --- | --- |
| This patient’s right to choose MAiD |  |  |  |  |  |  |
| Assisting a physician or NP to administer MAiD to this patient, within the scope of my future practice |  |  |  |  |  |  |

Very Uncomfortable (VU), Somewhat Uncomfortable (SU), Neutral (N), Somewhat Comfortable (SC), Very Comfortable (VC), Unsure (U)

**Case Study 2: MAiD and mature minor**

You are working on a pediatric oncology unit and a 16 year old patient that you have been working with over the last three shifts, who is receiving treatment for terminal and incurable cancer, asks to receive MAiD.

| What is your comfort level with… | VU | SU | N | SC | VC | U |
| --- | --- | --- | --- | --- | --- | --- |
| This patient’s right to choose MAiD |  |  |  |  |  |  |
| Assisting a physician or NP to administer MAiD to this patient, within the scope of my future practice |  |  |  |  |  |  |

Very Uncomfortable (VU), Somewhat Uncomfortable (SU), Neutral (N), Somewhat Comfortable (SC), Very Comfortable (VC), Unsure (U)

**Case Study 3: MAiD when psychiatric illness is underlying condition**

You are working in an acute psychiatric unit and a client who was diagnosed ten years ago with severe depression and schizophrenia requests MAiD. The client has no other medical conditions. The client reports being no longer able to cope with their mental condition and reports daily suffering with no relief from medication.

| What is your comfort level with… | VU | SU | N | SC | VC | U |
| --- | --- | --- | --- | --- | --- | --- |
| This patient’s right to choose MAiD |  |  |  |  |  |  |
| Assisting a physician or NP to administer MAiD to this patient, within the scope of my future practice |  |  |  |  |  |  |

Very Uncomfortable (VU), Somewhat Uncomfortable (SU), Neutral (N), Somewhat Comfortable (SC), Very Comfortable (VC), Unsure (U)

**Case Study 4: MAiD using an advance request**

You are working with a client on the surgical unit who had a total hysterectomy for cervical cancer, and is now four days post-operative. A scan has found metastases on the patient’s brain. The patient makes an advance request for MAiD, fearing they will lose capacity to make a decision once the metastases progress.

| What is your comfort level with… | VU | SU | N | SC | VC | U |
| --- | --- | --- | --- | --- | --- | --- |
| This patient’s right to choose MAiD in advance |  |  |  |  |  |  |
| Assisting a physician or NP to administer MAiD to this patient based on a request in advance, within the scope of my future practice |  |  |  |  |  |  |

Very Uncomfortable (VU), Somewhat Uncomfortable (SU), Neutral (N), Somewhat Comfortable (SC), Very Comfortable (VC), Unsure (U)

**Part 7. Respondent Demographics**

*Note: In small groups or samples in which demographic data could be identifying, such as a single cohort of students, demographic variables should be eliminated or reduced to ensure anonymity.

Please answer the following questions about yourself:

1. My age in years is
2. Less than 20
3. 20-29
4. 30-39
5. 40-49
6. 50-59
7. 60 or older
8. I identify as
9. Female
10. Male
11. Non-binary
12. Prefer not to say
13. Prefer to self-describe (textbox for this selection)
14. I was primarily raised in [Country List Pull Down]*
15. If Canada, in what province/territory were you raised?*[Province/Territory Pull Down]
16. How important is your religion to you?
    1. Very unimportant
    2. Unimportant
    3. Neutral
    4. Important
    5. Very important
    6. Prefer not to say
17. How important is spirituality to you?
    1. Very unimportant
    2. Unimportant
    3. Neutral
    4. Important
    5. Very important
    6. Prefer not to say
